# Assessment of Microbiological Safety of Water in Public Swimming Pools in Guangzhou, China

**DOI:** 10.3390/ijerph15071416

**Published:** 2018-07-05

**Authors:** Xiaohong Wei, Juntao Li, Shuiping Hou, Conghui Xu, Hao Zhang, Edward Robert Atwill, Xunde Li, Zhicong Yang, Shouyi Chen

**Affiliations:** 1 Guangzhou Center for Disease Control and Prevention, Guangzhou 510440, China; weijuanwo@163.com (X.W.); jtwl1011@163.com (J.L.); gzcdc367@163.com (S.H.); yeast-27@163.com (C.X.); haozsu@163.com (H.Z.); 2 School of Public Health, Sun Yat-Sen University, Guangzhou 510030, China; 3 Department of Population Health and Reproduction, University of California Davis, California, CA 95616, USA; ratwill@ucdavis.edu (E.R.A.); xdli@ucdavis.edu (X.L.); 4 Western Institute for Food Safety and Security, University of California Davis, California, CA 95616, USA

**Keywords:** swimming pool, water, *Giardia*, *Cryptosporidium*, *P. aeruginosa*, antibiotic resistance, multi-locus sequence typing

## Abstract

This study assessed microbiological safety of water from public swimming pools in Guangzhou, China. Water samples from 39 outdoor municipal swimming pools were collected from late June to early September, 2013 and subjected to detection of protozoa (*Giardia* and *Cryptosporidium*) and bacteria (*Pseudomonas aeruginos*, total coliforms, *E. coli*, *E. coli* O157, *Shigella*, and *Salmonella*). *Cryptosporidium* and *Giardia* were both detected in 5 (12.8%) swimming pools. Total coliforms were detected in 4 (10.3%) samples with concentrations ranging from 1.3 to 154.0 MPN/100 mL while *E. coli* was detected in 4 (10.3%) samples with concentrations ranging from 0.5 to 5.3 MPN/100 mL. *P. aeruginosa* was detected in 27 (69.2%) samples but *E. coli* O157, *Shigella* and *Salmonella* were not detected. Among these swimming pools, 9 (23%) met the Chinese National Standard of residual chlorine levels and 24 (62%) were tested free of residual chlorine at least once. The multi-locus sequence typing (MLST) analysis showed that all *P. aeruginosa* isolates belonged to new sequence types (STs) with dominant ST-1764 and ST-D distributed in different locations within the area. Some *P. aeruginosa* strains were resistant to medically important antibiotics. Results indicate potential public health risks due to the presence of microbiological pathogens in public swimming pools in this area.

## 1. Introduction

Many microbiological pathogens, including bacteria, viruses, and parasites can cause waterborne disease [1]. Waterborne disease is estimated to cause more than 2.2 million deaths per year and many cases of illness every day, including diarrhea, gastrointestinal diseases and other systemic illnesses [2,3]. Waterborne disease can have a significant impact locally and globally [4]. Waterborne infections are transmitted in numerous ways, including but not limited to ingestion, airborne or contact with contaminated water by a variety of infectious agents.

In addition to drinking water, which serves as a route for waterborne illness worldwide, especially in developing countries [5], exposure to recreational water also frequently causes outbreaks of waterborne diseases in both developed and developing countries [6,7,8,9]. For examples of recreational water related outbreaks in developed countries, there have been reports of waterborne disease associated with swimming pools in Australia [10], England and Wales [11] and other countries [12]. Most countries have programs for monitoring microbiological safety of swimming pool water. However, diverse public health and economic conditions among countries result in different microbiological and physical-chemical standards for swimming pool water even within countries of the European Union [13]. In China, a National Standard for swimming pool water quality, entitled “Hygienic Standard for Swimming Places” has been established (GB9667-1996). Major parameters in the National Standard are water temperature (22–26 °C), pH (6.5–8.5), turbidity (≤5 NTU), urea (≤3.5 mg/L), free residual chlorine levels (0.3–0.5 mg/L), total bacteria count (≤1000 cfu/mL), total coliforms (≤18 cfu/L). Although some recent works have studied water quality of swimming pools in China, most of these studies have primarily focused on the basic parameters that are highlighted in the National Standards, including pH, turbidity, urea content and total coliforms [14,15,16]. Literature on the microbiological safety of swimming pool water in China has been notably sparse. Furthermore, from a public health perspective it remains unknown if swimming pool water transmits antibiotic resistant bacteria. By detection and analysis of key waterborne protozoa and bacteria, this study evaluated the microbiological safety of water in outdoor public swimming pools in Guangzhou, which is a large metropolitan area in southern China.

## 2. Materials and Methods

### 2.1. Swimming Pools Selection and Water Sample Collection

This survey was conducted between 20 June and 4 September 2013. Due to the potential differences in public health conditions between suburban and urban districts within the Guangzhou area [17], the suburban Baiyun district and the urban Haizhu district were chosen for sampling. In total 39 pools, including 27 pools from the Baiyun district (21 residential pools, 6 pools from two water parks) and 12 pools from the Haizhu district (5 residential pools, 3 school pools, and 4 pools from one water park) were enrolled in the study. All enrolled pools were outdoor public swimming pools. Three swimming pools were exclusively for children while the remaining 36 pools were for both adults and children. Water samples were collected between 10:00 a.m. and noon, a period prior to the pools being open to swimmers. Using a submersible pump, 50 L water samples were pumped 10 cm from the bottom of the pool into two sterile 25 L carboys. Four hundred milligrams of sodium thiosulfate (Na_2_S_2_O_3_, XK13-001-00008, GB/T-637 standard, Guangzhou, China) was added to each carboy for dechlorination of water. Carboys were placed in containers with ice and transported to the laboratory within two hours and stored in a cold room (4 °C) upon arrival at the laboratory and before processing.

A questionnaire was filled out for each swimming pool in order to support the microbiological analysis. The questionnaires contained three sections that include general data on the swimming pools (type, location, dimensions and depth), levels of residual chlorine, and management information (opening time, hours of operation, availability of swimming-suit rentals, whether toddlers were required to wear diapers during swimming, methods used to refresh water, frequency of testing residual chlorine levels).

### 2.2. Detection of Microbiological Pathogens from Water

With the aid of hollow fiber ultrafiltration as described by Rhodes et al. [18], the 50 L water sample was filtered no later than 24 h after collection and concentrated to approximately 700 mL (retentate). To test for *Giardia* and *Cryptosporidium*, 500 mL of the retentate was centrifuged at 4000 rcf at 25 °C for 15 min without breaking. Supernatant was discarded by aspiration and approximately 3 mL of residual pellet was harvested. An immunomagnetic separation (IMS) procedure was carried out to separate (oo) cysts from concentrated water samples using the Dynabeads GC-Combo kit (Invitrogen Dynal AS, Oslo, Norway) according to the manufacturer’s instructions. The IMS was carried out using a Bead Retriever with the procedure of ‘GC Combo’ (Invitrogen, Finland). Immunofluorescent staining of final IMS products (approximately 50 μL) was performed using the Aqua-Glo G/C Direct kit (Waterborne, New Orleans, LA, USA) according to the manufacturer’s instructions. Slides were examined for (oo) cysts using an immunofluorescent microscope (Leica DM6000B).

All bacteria were detected according to China’s National Standard (GB) protocols. Specifically, *P. aeruginosa* was detected using methods described in the Hygienic Standard for Cosmetics (GB7918.4-2007, Ministry of Health, China). The methods used for detection of other bacteria were based on the National Food Safety Standard (Ministry of Health, China), i.e., GB4789.3-2010 for total coliforms, GB4789.38-2012 for *E. coli*, GB4789.36-2008 for *E. coli* O157, GB4789.5-2012 for *Shigella* and GB4789.4-2010 for *Salmonella*, respectively. The procedures for detection of these bacteria are described below.

To detect *P. aeruginosa*, 10 mL of retentate was resuspended in 90 mL of Soya Casein Digest Lecithin Polysorbate (SCDLP) broth and incubated at 37 °C for 24 h. Then, 100 µL of the culture was streaked on cetrimide agar and incubated at 37 °C for 24 h. For total coliforms, triplication of serially diluted retentate (1, 0.1 and 0.01 mL) were resuspended in tubes of LST (Lauryl Sulfate Tryptose) broth and incubated at 37 °C for 48 h. The most probable number (MPN) was calculated according to the number of tubes that generated positive reactions (generating bubbles). Similarly, triplication of serially diluted culture (1, 0.1, and 0.01 mL) of LST broth generating bubbles were transferred into *E. coli* broth and incubated at 44.5 °C for 48 h for calculating MPN of *E. coli*. To detect *E. coli* O157, 25 mL of retentate was added to 225 mL of modified *E. coli* broth and incubated at 37 °C for 24 h. An aliquot of the culture was streaked onto *E. coli* O157 chromogenic medium and incubated at 37 °C for 24 h. Red or purple colonies were chosen to cultivate on TSI (Triple Sugar Iron) agar medium at 37 °C for 24 h. For detection of *Shigella*, 25 mL of retentate was added to 225 mL of *Shigella* broth and incubated at 42 °C for 20 h. Then, an aliquot of the culture was streaked onto *Shigella* chromogenic medium and incubated at 37 °C for 48 h. Typical colonies were selected for cultivation on TSI (Triple Sugar Iron) agar medium at 37 °C for 24 h. For detection of *Salmonella*, 25 mL of retentate was added to 225 mL of Buffered Peptone Water (BPW) and incubated at 37 °C for 18 h. An aliquot of the BPW culture was added to 10 mL of TTB (Tatrathionate Broth) and incubated at 42 °C for 24 h. The culture in TTB was then streaked onto *Salmonella* chromogenic medium and incubated at 37 °C for 24 h. Purple or red colonies were selected for incubation on TSI (Triple Sugar Iron) agar medium at 37 °C for 24 h. Isolates of *P. aeruginosa*, *Shigella* and *Salmonella* were confirmed biochemically and phenotypically using the VITEK2 GN method (bioMériux, Marcy d’Etoile, France).

### 2.3. Testing P. aeruginosa Susceptibility to Antibiotics

*P. aeruginosa* susceptibility to antibiotics was tested using the VITEK2 AST-GN04 method (bioMériux, Marcy d’Etoile, France). The selection of antimicrobial agents and interpretation of minimum inhibitory concentration (MIC) (S = susceptible, IR = intermediate resistant, R = resistant) were based on the Clinical and Laboratory Standards Institute [19]. The antimicrobial agents tested are of medical importance, including piperacillin, piperacillin-tazobactam, ticarcillin-clavulanic acid, ceftazidime, cefepime, imipenem, gentamicin, tobramycin, amikacin, levofloxacin and ciprofloxacin.

### 2.4. Multilocus Sequence Typing (MLST) of P. aeruginosa

Two or three typical *P. aeruginosa* colonies were picked and homogenized in 100 μL autoclaved deionized water. The bacterial solutions were incubated at 100 °C for 10 min followed by cooling at 4 °C for 3 min, then centrifuging at 12,000× *g* for 3 min. The primer pairs used for PCR and sequencing are shown in Table 1. The amplification reactions and conditions for MLST of *P. aeruginosa* were performed as described in the PubMLST (http://pubmlst.org/paeruginosa/info/primers.shtml). Because the original temperature could not successfully amplify mutL, ppsA and trpE alleles, slightly modified conditions (primer annealing temperature) were used to optimize amplification in each reaction. The following adjusted PCR protocol was used: denaturing at 95 °C for 1 min, 35 cycles of 1 min at 95 °C (denaturing), 1 min at 55–57 °C (primer annealing, 57 °C for mutL and ppsA, 55 °C for trpE) and 1 min at 72 °C (primer extension), and a final elongation step at 72 °C for 10 min. Sequencing of all the genes were performed by Life Biological Technology Company (Shanghai, China). Sequences from the study and alleles and STs from the PubMLST (http://pubmlst.org/) were used to construct a phylogenetic tree of *P. aeruginosa* isolates using MLST Data Analysis-Tree drawing (https://pubmlst.org/paeruginosa/). A population snapshot was operated using the software eBRUST V3 available at the website of http://eburst.mlst.net/v3/enter_data/comparative/.

### 2.5. Statistical Analysis

The Chi-square (χ^2^) test was applied to assess the potential differences in *Giardia* and *Cryptosporidium* presence in water between suburban and urban swimming pools, because we found the prevalence of *Cryptosporidium* was significantly higher in children hospitalized in suburban hospitals than in urban hospitals in the same area in a previous study [17]. Logistic correlation was applied to analyze whether the presence of *P. aeruginosa* was associated with the levels of free residual chlorine in water.

## 3. Results

### 3.1. Characteristics of Swimming Pools

Outdoor swimming pools in the Guangzhou area mostly open between June and September. The swimming water of all enrolled pools is refreshed by refilling the pools with tap water as needed. No swimming suit rental was available in any of the surveyed swimming pools. Swimmers brought their own swimming suits and children were not required to wear diapers when swimming. The studied swimming pools had a diversity of shapes and surface areas that ranged from 100 to 1000 m^2^. The depth of these pools ranged from 0.3 m to 2.0 m with 1.0 to 1.3 m for the majority of the enrolled pools. All swimming pools used standard chlorination to disinfect water, and no further treatment of the water had taken place. Among the pools, 24 (62%) checked free residual chlorine levels once per day; 8 (20%) check twice per day; and 7 (18%) check 3–5 times per day. Free residual chlorine levels were >0.5 mg/L in 18 (46%) swimming pools and <0.3 mg/L in 12 (31%) of the swimming pools. The highest free chlorine level was 2.0 mg/L and the lowest free chlorine level was 0 mg/L. According to the Chinese National Standard, ‘Hygienic Standard for Swimming Places’ (GB9667-1996, China) the free residual chlorine level in swimming water should be between 0.3 mg/L and 0.5 mg/L. Our survey results showed that only 9 (23%) of the studied swimming pools had free residual chlorine levels that met this criteria.

### 3.2. Occurrence of Protozoa in Swimming Pool Water

*Cryptosporidium* and *Giardia* were both detected in 12.8% (5/39) of the enrolled swimming pools. Among these positive pools, 2 pools were positive for *Giardia* only, another 2 were positive for *Cryptosporidium* only, and 1 was positive for both *Giardia* and *Cryptosporidium*. Concentrations of (oo) cysts in positive pool water were 0.03 cysts/L of *Giardia* and 0.03–0.14 oocysts/L of *Cryptosporidium*. The proportional occurrences of both *Giardia* and *Cryptosporidium* were 25% (3/12) and 7.4% (2/27) in pools in the Baiyun district (suburban) and the Haizhu district (urban), respectively. There was no significant difference in the occurrence of *Giardia* and *Cryptosporidium* in swimming pools between suburban and urban areas (*p* = 0.159).

### 3.3. Occurrence of Bacteria in Swimming Pool Water

Total coliforms were detected in 4 (10.3%) of 39 swimming pools, with concentrations of 1.3, 3.2, 154.0, and 154.0 MPN/100 mL, respectively. Concentrations of total coliform in 2 of the positive pools were lower while that of the other 2 pools were higher than the 18 cfu/L specified in the Chinese Standard. *E. coli* was also detected in the same 4 (10.3%) of 39 swimming pools, with concentrations of 0.5, 1.3, 3.2, and 5.3 MPN/100 mL. Using the standard methods stated above, pathogenic *E. coli* O157, *Shigella* and *Salmonella* were not detected in any of the enrolled swimming pools. *P. aeruginosa* was detected in 27 (69.2%) of the swimming pools. Based on the Chinese National Standard, the free residual chlorine levels in water of these swimming pools were divided into three groups: <0.3 mg/L, 0.3–0.5 mg/L, and >0.5 mg/L. The proportional occurrence of *P. aeruginosa* in each group is shown in Table 2. As shown in the table, the results of logistic correlation analysis using <0.3 mg/L as the control group indicated that there was no significant relationship between the level of free residual chlorine and the presence of *P. aeruginosa*. (*p* = 0.55 and 1.00, respectively).

### 3.4. MLST of P. aeruginosa

**Figure 1:**
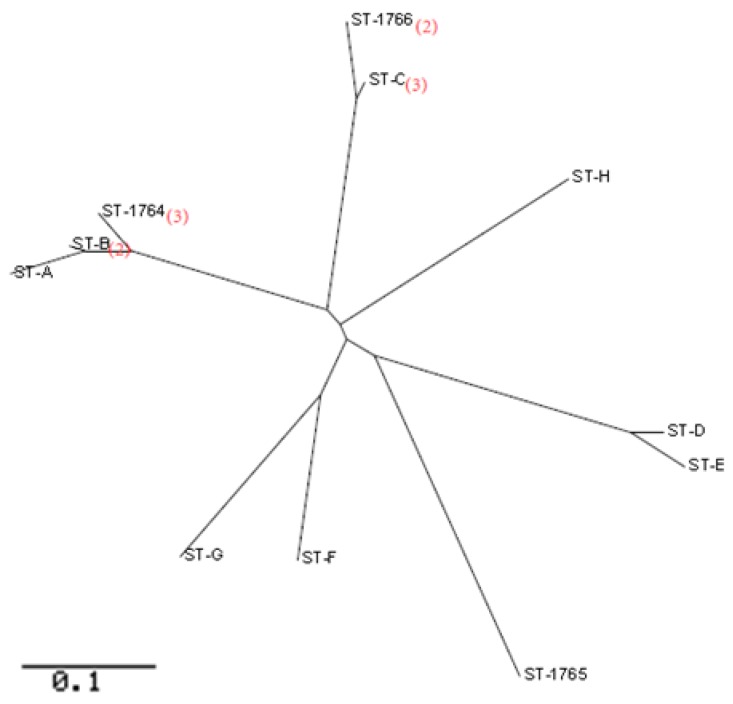
Phylogenetic tree of MLSTs of 17 *P. aeruginosa* isolates each from different swimming pools. The red digits in the parentheses denote the numbers of the STs. The number of unmarked STs was 1.

**Figure 2:**
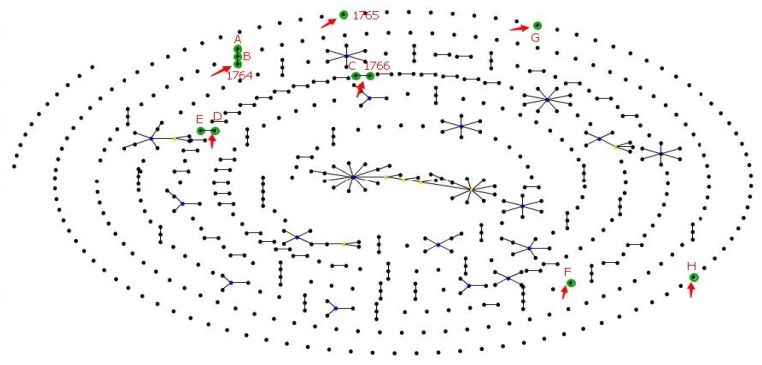
Population structure of the 1833 STs listed in the *P. aeruginosa* PubMLST database and the 11 STs from this study. Note: The figure shows all BURST groups (connected STs), singleton STs, ancestral founders (blue STs), and subgroup founders (yellow STs). Dots represent STs and lines connect single-locus variants. Purple letters and digits represent STs of *P. aeruginosa* from the current study and STs with green halos represent STs detected only in the current study. Line length and singleton ST placement is arbitrary.

Among the 27 *P. aeruginosa* isolates, 17 isolates were positive for all the seven housekeeping genes, therefore they were analyzed using MLST. One or more housekeeping genes in each isolate could not be amplified or sequenced, as a result, 6 alleles of acsA, ppsA and trpE; 5 alleles of aroE, mutL and nuoD; and 7 alleles of guaA were successfully sequenced (Table 3). The MLST analysis identified 11 STs from the tested isolates, all were distinct from existing STs at (http://pubmlst.org/) and therefore were novel STs. Using the curator Ellie Pinnock of PubMLST (http://pubmlst.org/), three ST types have been named as ST-1764, ST-1765 and ST-1766 and the remaining types are designated as ST-A, ST-B, ST-C, ST-D, ST-E, ST-F, ST-G and ST-H. According to the most stringent definition by the eBURST, a ST group was defined as isolates sharing identical alleles at ≥6 of the 7 loci among isolates in a group that belonged to a clonal complex. Otherwise it was singleton. Isolates stemming from a group were considered as belonging to a single clonal complex. The STs with one and two distinct loci compared to the founder strain were named single-locus variants (SLV) and double-locus variants (DLV), respectively (Table 3). Phylogenetic analysis indicated that these STs from different pools and different districts fell into the same branches with the exceptions of ST-D and ST-E in one branch, ST-C and ST-1766 in one branch, and ST-A, ST-B, and ST-1764 in one branch (Figure 1). The type and locations of these samples are listed in Table 3. To further explore the associations between the *P. aeruginosa* isolates from current study and *P. aeruginosa* isolates from the database of PubMLST, a population structure of *P. aeruginosa* isolates was created (Figure 2). As shown in the figure, STs and isolates of *P. aeruginosa* from the current study were distinct from the 1833 STs at the PubMLST, which supports the assertion of the existence of unique and novel STs of *P. aeruginosa* in swimming pool water in the Guangzhou area.

### 3.5. P. aeruginosa Susceptibility to Antibiotics

Susceptibility tests were performed on all the 27 *P. aeruginosa* isolates, each from different pools. Six isolates (ID# 2, 16, 17, 32, 33 and 37) exhibited different levels of susceptibility (R, IR, or S) to tested antibiotics while all the other isolates (IDs not provided) were susceptible to all tested drugs (Table 4). Among the tested drugs, 2 isolates (#2 and #37) were resistant to piperacillin, 2 isolates (#17 and #33) were resistant to imipenem, and 1 isolate (#17) was resistant to gentamicin (Table 4). The isolates #2, #16, #17 and #37 corresponded to the MLST groups of ST-A, ST-1765, ST-1766, and ST-D, respectively. All the rest of the isolates (not listed in Table 4) were susceptible to all tested drugs.

## 4. Discussion

According to our observations during the survey and the survey results, overall there is a lack of good practices in pool management, including water disinfection. The free residual chlorine levels of 18 (46%) swimming pools were greater than 0.5 mg/L, which is higher than that of China’s National Standard for swimming pools. However, the standards for free residual chlorine for swimming pool water in some European countries [13] are higher than the Chinese standard, which may increase the efficiency of water disinfection. On the other hand, the pools open in June through September which is the warmest season of the whole year in Guangzhou. UV radiation can lead to the formation of chlorate in the water, which reduces the concentration of free chlorine available in water and subsequently impacts the effects of chlorine disinfection. Also, because children were not required to wear diapers when swimming, there was a potential of pool water contamination by children with diarrhea, which is a reported route for swimming pool contamination with *Cryptosporidium* [20]. Finally, the manner of refreshing pool water (adding water when necessary instead of replacing water completely) could facilitate the accumulation of pathogens in water.

The overall prevalence of *Giardia* and *Cryptosporidium* were both 12.8% in swimming pools during the study period. No significant differences in the occurrence of the two protozoal parasites were found between pools in urban and suburban areas. This is converse to our previous findings that prevalence of *Cryptosporidium* was significantly higher in children hospitalized in suburban hospitals than in urban hospitals in the same area [17]. This is probably because of the different transmission routes of *Cryptosporidium* in children patients living in urban and suburban areas. Due to limited numbers of (oo) cysts detected from pool water we did not characterize the two parasites by genotyping, hence the genotypes and zoonotic potential of the two parasites in pool water were unknown. In our previous study we found that the dominant species of *Cryptosporidium* in children hospitalized for diarrhea in the same area was *C. parvum*, a species that infects humans and a wide range of animals [17]. Also, because of the limited numbers of oocysts detected in samples, we did not assess the viability of oocysts, therefore, it was unknown if the oocysts detected in pool water were infective. However, it is known that oocysts are resistant to chlorine and cause waterborne illness associated with swimming pools [21,22]. *Cryptosporidium* and *Giardia* are infectious to people, especially children, the elderly, others with weakened immunity (such as AIDS patients) and travelers, and may cause severe diarrhea and even death [23,24]. The two parasites are major causes of waterborne parasitic infections all over the world, including developed countries such as New Zealand, Australia and USA [25]. A challenge to control these waterborne parasites is that chlorination is ineffective against (oo) cysts [25]. Although UV irradiation [26] and ozone [27] show promising effects against (oo) cysts, these methods have not been practical for disinfection of swimming pool water. As a result, waterborne *Cryptosporidium* and *Giardia* associated with swimming occur frequently [28,29]. Therefore, it was not a surprise to detect *Cryptosporidium* and *Giardia* in swimming pool water in the area, and the results demonstrated that control of the two most common waterborne protozoa is a continuous task in Guangzhou area, as it is elsewhere.

With respect to bacterial pathogens, both total coliform and *E. coli* were detected in approximately 10% of the enrolled swimming pools. These indicator organisms can potentially cause various diseases, particularly in children under five years old and the elderly [6]. Presence of these organisms in water often indicates the ineffectiveness of water disinfection [30], especially the pools with total coliform concentrations higher than the National Standard in this study. The good news is that pathogenic bacteria, *E. coli* O157, *Shigella* and *Salmonella* were not detected in all swimming pools, including those 10% of pools that were positive for total coliforms and *E. coli*. However, another pathogenic bacterium, *P. aeruginosa* was found to be highly prevalent (69%) in studied swimming pools.

*P. aeruginosa* is ubiquitous in water and is resistant to chemical disinfectants such as chlorine [31]. *P. aeruginosa* cause a variety of disease including folliculitis, external otitis, keratitis, urinary infections and gastrointestinal infections through exposure to skin, ears, eyes, urinary track, lungs and the gut [12,30,32]. Seventy-nine percent of ear infections in swimmers were ascribed to *P. aeruginosa* with symptoms ranging from earache to hearing loss [33]. According to a survey of waterborne diseases in the USA between 1999 and 2008, *P. aeruginosa* was the second most common waterborne pathogenic micro-organism after *Cryptosporidium* [6]. A study reported that *P. aeruginosa* was detected in 95 (59%) of 161 samples of swimming pool filter backwash in Atlanta, Georgia, GA, USA [34]. In the present study we detected similarly high occurrence (69%) of *P. aeruginosa* in public swimming pools in the Guangzhou area. Also, the same STs or the same group of STs of *P. aeruginosa* were distributed among different types of pools in different complexes, and in different locations (Table 3). Our MLST (Figure 1) and population structure analysis (Figure 2) showed that STs of *P. aeruginosa* from swimming pools in the studied area were distinct from strains from other areas and other types of samples, indicating that unique *P. aeruginosa* strains were present in the aquatic environment in the area. Although our study did not determine the routes of transmission of *P. aeruginosa* in the area, *P. aeruginosa* can be carried on the equipment of swimmers [33]. Finally, several isolates of *P. aeruginosa* from swimming pools were resistant to some medically important antibiotics such as piperacillin, imipenem, and gentamicin (Table 4). This result is consistent with reports that *P. aeruginosa* isolates from water environments have lower antibiotic resistance than those of clinical specimens [35]. However, the presence of antibiotic resistant *P. aeruginosa* in swimming pool water indicates that swimming pool water can serve as a route for transmitting antibiotic resistant bacteria and genes, hence presenting additional risks to swimmers who use public swimming pools and workers who perform maintenance of the pools.

Waterborne illness associated with swimming pools is commonly attributed to fecal contamination of water either by swimmers or animals that have access to outdoor pools [36,37]. Other non-fecal substances, such as vomit, mucus, saliva and skin can also serve as sources of pathogenic micro-organisms in swimming pools [38]. Although there are microbiological standards using total bacteria count and total coliform for swimming water, research on microbiological safety associated with pathogens in swimming pool water has been sparse in China. Our study showed the presence of pathogens such as *Cryptosporidium*, *Giardia* and antibiotic resistant *P. aeruginosa* in public swimming pools that potentially pose risks to public health. Data from our study and future similar studies will provide a background for public health agencies and communities to improve the management of public swimming pools in order to prevent waterborne illness associated with swimming pools.

## 5. Conclusions

This is the initial work of assessing microbiological safety of water in public swimming pools in Guangzhou, China. The presence of *Cryptosporidium*, *Giardia* and antibiotic resistant *P. aeruginosa* in swimming water indicate potential risks to public health in this area. Results suggest the need of updating management of swimming pool facilities and treatment of swimming water in order to prevent waterborne illness associated with swimming in the area.

## Figures and Tables

**Table 1:** Primers used for multi-locus sequence typing (MLST) of *P. aeruginosa* isolates.

Locus and Functions		Primers Sequence (5′—3′)		Size (bp)
		Forward	Reverse	
acsA	Amplification	ACCTGGTGTACGCCTCGCTGAC	GACATAGATGCCCTGCCCCTTGAT	842
	Sequencing	GCCACACCTACATCGTCTAT	GTGGACAACCTCGGCAACCT	390
aroE	Amplification	TGGGGCTATGACTGGAAACC	TAACCCGGTTTTGTGATTCCTACA	825
	Sequencing	ATGTCACCGTGCCGTTCAAG	TGAAGGCAGTCGGTTCCTTG	495
guaA	Amplification	CGGCCTCGACGTGTGGATGA	GAACGCCTGGCTGGTCTTGTGGTA	940
	Sequencing	AGGTCGGTTCCTCCAAGGTC	TCAAGTCGCACCACAACGTC	372
mutL	Amplification	CCAGATCGCCGCCGGTGAGGTG	CAGGGTGCCATAGAGGAAGTC	940
	Sequencing	AGAAGACCGAGTTCGACCAT	ATGACTTCCTCTATGGCACC	441
nuoD	Amplification	ACCGCCACCCGTACTG	TCTCGCCCATCTTGACCA	1042
	Sequencing	ACGGCGAGAACGAGGACTAC	TTCACCTTCACCGACCGCCA	366
ppsA	Amplification	GGTCGCTCGGTCAAGGTAGTGG	GGGTTCTCTTCTTCCGGCTCGTAG	989
	Sequencing	GGTGACGACGGCAAGCTGTA	TCCTGTGCCGAAGGCGATAC	369
trpE	Amplification	GCGGCCCAGGGTCGTGAG	CCCGGCGCTTGTTGATGGTT	811
	Sequencing	TTCAACTTCGGCGACTTCCA	GGTGTCCATGTTGCCGTTCC	441

**Table 2:** Occurrence of *P. aeruginosa* in swimming pool water with different residual levels of free chlorine.

Residual Levels of Free Chlorine (mg/L)	% (Positive/Total) Occurrence of *P. aeruginosa*	*p*	OR (95% CI)
<0.3	66.7 (8/12)	-	-
0.3 to 0.5	77.8 (7/9)	1.00	1.00 (0.21, 4.71)
>0.5	66.7 (12/18)	0.55	1.75 (0.28, 11.15)
Total	69.2 (27/39)	-	-

Note: OR = odds ratio, CI = confidence interval. <0.3 mg/L was used as control group.

**Table 3:** List of the alleles and bioinformatics analysis of STs of *P. aeruginosa* isolates from public swimming pools in Guangzhou, 2013.

acsA	aroE	guaA	mutL	nuoD	ppsA	trpE	Group	STs	SLV	DLV	Freq	Pool Type	District
81	11	112	5	73	20	139	1	ST-A	1	1	1	Residential	Haizhu
81	11	112	5	13	20	139	1	ST-B	2	0	2	Residential	Baiyun
81	11	57	5	13	20	139	1	ST-1764	1	1	3	Residential	Baiyun
5	3	95	5	93	6	47	2	ST-1766	1	0	2	Residential; Water park	Baiyun; Haizhu
5	3	95	5	13	6	47	2	ST-C	1	0	3	Residential; Water park *	Baiyun; Haizhu
70	5	72	2	3	20	26	3	ST-D	1	0	1	School	Haizhu
70	5	72	2	3	4	26	3	ST-E	1	0	1	Water park	Baiyun
5	5	57	13	13	74	3	Singleton	ST-F	-	-	1	Water park	Haizhu
83	5	9	3	13	10	3	Singleton	ST-G	-	-	1	Residential	Baiyun
32	13	24	13	13	6	25	Singleton	ST-H	-	-	1	Water park	Baiyun
39	6	4	14	61	15	2	Singleton	ST-1765	-	-	1	Residential	Baiyun

* Represents two pools from the water park located in Haizhu district.

**Table 4:** Phenotypic antibiotic resistance traits of *P. aeruginosa* from public swimming pools in Guangzhou, 2013.

Isolates ID	PENICILLINS	Β-LACTAM		Cephems		Carbapenems	Aminoglycosides			Fluoroquinolones	
	Piperacillin	Ticarcillin-Clavulanic Acid	Tazobactam	Ceftazidime	Cefepime	Imipenem	Gentamicin	Tobramycin	Amikacin	Ciprofloxacin	Levofloxacin
2	R *	S	S	S	S	S	S	S	S	S	S
16	IR **	S	S	S	S	S	IR	S	S	S	S
17	S ***	S	S	S	S	R	R	S	S	S	S
32	IR	S	S	S	S	S	IR	S	S	S	S
33	S	S	S	S	S	R	S	S	S	S	S
37	R	S	S	S	S	S	S	S	S	S	S
All other isolates	S	S	S	S	S	S	S	S	S	S	S

* R = Resistant; ** IR = Intermediate Resistant; *** S = Susceptible.
